# Building Primary Health Care Personnel’s Support for a Patient Portal While Alleviating eHealth-Related Stress: Survey Study

**DOI:** 10.2196/28976

**Published:** 2021-09-22

**Authors:** Iiris Hörhammer, Sari Kujala, Pirjo Hilama, Tarja Heponiemi

**Affiliations:** 1 Department of Industrial Engineering and Management Aalto University Espoo Finland; 2 Department of Computer Science Aalto University Espoo Finland; 3 Social Health Care Joint Authority of South-Savo Mikkeli Finland; 4 Social and Health System Research Unit National Institute for Health and Welfare Helsinki Finland

**Keywords:** patient portal, implementation, adoption, health care personnel, eHealth-related stress

## Abstract

**Background:**

Health care personnel’s (HCP) engagement in patient portal implementation is necessary in embedding the use of the portal in everyday practices of a health care organization. While portal implementation may raise personnel’s positive expectations of the benefits in patient care, it is often also stressful for them due to increased workloads and disruptions in clinical workflows. An understanding of social and technical factors that build personnel’s support for patient portal implementation and alleviate their eHealth-related stress is therefore needed to realize the full potential of portals.

**Objective:**

The aim of this study was to explore the influence of managerial implementation practices, information technology (IT) usability, and personnel’s eHealth competences on support for patient portal implementation and eHealth-related stress among primary HCP.

**Methods:**

The data were collected through a survey of 919 members at 2 health organizations in Finland. Linear and logistic regression models were fitted to study the associations between the variables.

**Results:**

Professionals’ eHealth competence (*β*=.15, *P*<.001), usability (*β*=.11, *P*<.001), and implementation practices (*β*=.07, *P*<.001) were positively associated with professionals’ support and negatively associated with professionals eHealth-related stress (*β*=−.07, *P*=.010; *β*=−.27, *P*<.001; and *β*=−.14, *P*<.001, respectively). Professionals’ support was associated with their promotion of the portal to the patients (odds ratio 1.22, 95% CI 1.07-1.40).

**Conclusions:**

The adoption of appropriate implementation practices and the usability of the technology can build personnel’s support for a patient portal and alleviate their stress related to eHealth. Personnel’s support is manifested in their promotion of the portal to patients. Health care managers are encouraged to consider the usability of the technology and the good implementation practices, such as proper informing, engagement of the personnel in planning the services, and allocation of resources to improve eHealth competence, as prerequisites for meaningful and sustainable use of patient portals.

## Introduction

### Background

Patient portals provide online access to personal health information and interactions with health care providers. Thus, they can support patient self-management and enhance shared decision making [1-3]. However, as a precondition to the benefits of patient portals, studies have emphasized the importance of substantial initial investments required of health care organizations in the implementation [4-6]. Management must embed the use of portals in daily clinical practice to support their meaningful and sustained use [6]. Success of the implementation ultimately relies on how the health care personnel (HCP), together with the patients, use the portal [6,7]. HCP’s effort in making sense of the portal utility together with colleagues and patients is necessary for the normalization of the use [7-9]. Local champions persuade their peers and patients of the “professional appropriateness” of the new services [7]. While HCP’s endorsement of patient portals is critical for the adoption and continued use by the patients [4,10,11], studies [12,13], even in controlled pilot settings [14], have identified the failure to engage frontline employees in the promotion of digital tools.

Management and technical factors may explain inadequate engagement among personnel. Poor managerial practices [15,16] and technical difficulties [4,10] have been shown to inhibit health care professionals’ support. By contrast, good usability [6,15,17] and implementation practices, such as involving personnel in planning [18], and supporting the effective use of portals in clinical work [5,19-21], are essential to the successful adoption of health information technology (HIT). A study of the preimplementation phase of a patient portal found an association between health care professionals’ expectations of their organization’s implementation practices and their support for the portal [15]. However, little is known about this association in the postimplementation phase or whether personnel’s support for the portal is manifested in their collaborations with patients.

Most studies have discussed the opportunities provided by patient portals; however, few have focused on their adverse effects on personnel and their work. The adoption of new information technology (IT) has raised concerns among health care professionals regarding increased workloads and disrupted clinical workflows [22,23]. A recent study in Finland found that poorly functioning, time-consuming, and inadequate information systems have emerged as a significant source of stress in HCP’s work, and that stress related to information systems had increased between 2006 and 2015 [24]. Stress has been described to appear in a relationship between a person and the environment that is appraised as important for an individual but exceeds their coping resources [25]. While excessive stress experienced by HCPs may lead to decrease in their well-being, dissatisfaction at work, and even intent to leave the organization [26], the upward trend in HIT-related stress raises concerns about the sustainability of the health care system. HIT usability deficiencies have been associated with HCP’s high stress related to information systems [27] and general self-rated stress [28]. However, little is known about the influence of the implementation practices of HIT on personnel’s eHealth-related stress.

Building on recent sociotechnical research on the technical and organizational factors in successful HIT adoption and use [7,29-31], this study investigated the associations of (1) HCP’s perception of *organizational implementation practices* and (2) *patient portal* usability with *their support* for the technology and their *eHealth-related stress*. Moreover, to identify the possible manifestation of HCP’s support through their *interactions with the patients*, the association between personnel’s support for and promotion of the portal was examined.

### Research Model

Organizational practices surrounding health technology implementation can be determinative of personnel’s successful adoption. Such practices include proper informing of the new services before implementation [15,32], engagement of the personnel in planning [15], adequate training [19-21], and enough time for learning [18]. Expectations of good implementation practices have been found to be associated with health care professionals’ support for eHealth in the preimplementation phase [15]. Therefore, we hypothesize that:

H1: HCP’s positive perception of the implementation practices in their unit is positively associated with their support for a patient portal.

Studies have shown associations between HCP’s perception of HIT usability and their acceptance of the technology [33-35]. Therefore, we hypothesize:

H2: Usability is positively associated with HCP’s support for a patient portal.

Usability has been shown to relate to the skills needed to use a new technology [33,36]. Therefore, we also propose that personnel’s eHealth skills are associated with their support for the portal. In order to use eHealth in a meaningful way, personnel need new competences, not only in computer skills and literacy but also in the application of HIT in patient interactions, and the promotion of the technology to facilitate patient self-management [10,37]. Our hypothesis is:

H3: HCP’s self-rated eHealth competences are positively associated with their support for a patient portal.

HCP’s endorsement has been deemed essential to patients’ adoption and continued use of digital health tools [10,11]. Personnel who support a portal might be more likely to promote its use to their patients. We therefore hypothesize:

H4: HCP’s support for patient portal is positively associated with their promotion of the portal to patients.

Studies have identified HIT usability problems that disrupt HCP’s routine work [38]. Usability issues have been associated with elevated stress related to information systems, which might be alleviated by experience in using the technology [24,27]. Therefore, we hypothesize the following:

H5: Usability is negatively associated with HCP’s eHealth-related stress and

H6: HCP’s self-rated eHealth competence is negatively associated with their eHealth-related stress.

In addition to personnel’s competences, organizational practices in the development and implementation, such as end-user involvement in system development and work-procedure planning, have been suggested as potential alleviators of IT-related stress [24]. We hypothesize that:

H7: HCP’s positive perception of the implementation practices in their unit is negatively associated with HCP’s eHealth-related stress.

## Methods

### Study Setting

The survey study was conducted in 2 Finnish regional health and social care authorities (Organizations A and B) in 2017. Both authorities are public social and health care providers serving populations of approximately 100,000 (Organization A) and 40,000 (Organization B). At the time of the survey, the organizations had already implemented eHealth services, and aimed to increase their usage.

The functionalities of the patient portals in both organizations were online appointment booking, access to personal medical records, patient-reported medical history, and electronic messaging with HCP. In Organization A, digital symptom questionnaires, self-management instructions, and remote health care appointments had recently become available to certain patient groups. In both organizations, different functionalities had been gradually adopted over the 13 years preceding the survey; however, the pace had accelerated during the preceding 5 years.

An invitation to the online survey was sent to HCP via work email. The survey was also introduced on the health and social care authorities’ intranet news page. No exclusion of a subgroup of employees was applied. Participation was anonymous; however, the respondents had an opportunity to win movie tickets and 2 wireless computer mice. The study protocol was reviewed and approved by Aalto University’s Ethical Review Board.

### Questionnaire

The questionnaire contained questions about demographics, support for the patient portal implemented by the regional health and social care authority, self-perceived eHealth competences, and HIT implementation practices in the respondent’s unit. Personnel were also asked about usability and stress related to IT and the patient portal (Multimedia Appendix 1). All items, except those related to demographics and stress measurement, were answered on a 5-point Likert scale ranging from 1 (fully disagree) to 4 (fully agree), with the fifth option (don’t know) omitted from the analysis.

*eHealth competences* were assessed with 8 statements from a previously used scale [37] encompassing the use of eHealth tools in the personnel’s work. The use of the *recommended implementation practices* was assessed through 4 previously used items [15,39] regarding the resources and information given to the personnel for adopting new services and opportunities to participate in the planning of new services. *Personnel’s support* for the patient portal was measured with 5 items from the previously validated I-SEE questionnaire [15,39-41]. The items address a respondent’s personal and perceived collective support by colleagues and supervisors for the patient portal implementation.

IT *usability* was measured with 4 items as in our previous study [15]. We used 3 items from the Usability Metric for User Experience (UMUX) scale concerning utility, ease of use, and frustration related to the IT [42], and added a fourth item on general user satisfaction. A previously used measure [43-45] was used to assess *eHealth- and information systems–related stress*. This measure has been developed in Finland when examining the health and well-being of physicians [24,44]. It has previously been associated with, for example, experience in using information systems, cognitive workload, distress, and electronic health record usability [27,44]. The participants were asked how often during the previous 3 months they had been distracted by, worried about, or stressed about information systems, or IT equipment or software. A third item about stress related to the patient portal (eHealth services) was added to satisfy the scope of the study. The answers were rated on a 5-point Likert scale ranging from 1 (never) to 5 (very often).

To assess the frequency of HCP’s *promotion of the portal to patients,* the participants were asked to choose one of the following options: “never,” “1-4 times,” “5-9 times,” and “10 or more times.” Because of the small proportion of responses in the categories other than “never,” the 3 other response categories were combined to create a variable related to the promotion of the portal to a patient at least once (Yes/No).

### Statistical Analysis

Descriptive statistics and reliability analyses were performed and mean sum scores were computed for all study variables (Table 1 and Multimedia Appendix 2). The scale reliabilities (Cronbach α) of the Likert scale measures were all over .70, and therefore at an acceptable level [46]. Although the lowest reliability of .71 for eHealth-related stress was not very high, it can still be considered acceptable due to its very short length with only 3 items [47]. The hypotheses were tested through regression analysis. Two linear regression models were fitted with (1) personnel’s support for the patient portal and (2) their eHealth-related stress as the dependent variable. Usability, eHealth competence, eHealth-related stress, age, gender, work experience, and organization (Organization A or B) were the independent variables. A logistic regression model was fitted to assess the association between personnel’s support for the portal and their promotion of the portal to patients. The model was adjusted for age, gender, work experience, and profession. We fitted the models stepwise and report estimates from the univariate models, multivariate model with independent variables of interest only, and multivariate model with adjustments. To test for multicollinearity, the variance inflation factors were calculated for the regression variables. All the variance inflation factors were lower than 1.7, thus indicating that multicollinearity was not a concern [48].

## Results

### Respondents

A majority of the respondents were women (800/919, 87.1%) and nurses or assistant nurses (589/919, 64.1%) with an average age of 44 years (SD 11.7) and 11.6 years (SD 10.2) of work experience in their current duties. The other personnel were secretaries, social workers, doctors, and psychologists or other therapists (Table 1). The 919 respondents comprised approximately 20% of all health and social care personnel in the target organizations. The respondents well represented the Finnish health and social care professionals in terms of gender and age.

**Table 1:** Descriptive characteristics of participants (N=919).

Characteristic			Value
**Gender, n (%)**			
	Man	98 (10.7)	
	Woman	800 (87.1)	
	Not reported	21 (2.3)	
Age in years, mean (SD)			44.6 (11.7)
**Profession, n (%)**			
	Nurse/midwife/public health nurse	310 (33.7)	
	Assistant/other nurse	279 (30.4)	
	Social worker	61 (6.6)	
	Ward/department secretary	55 (6.0)	
	Doctor/dentist	52 (5.7)	
	Psychologist/physiotherapist and other therapist	51 (5.6)	
	Administrator	45 (4.9)	
	Maintenance and technical support	41 (4.5)	
	Dental nurse or hygienist	13 (1.4)	
	Other	12 (1.3)	
**Organization, n (%)**			
	A	209 (22.7)	
	B	710 (77.3)	
Years of work experience in similar tasks, mean (SD)			11.6 (10.2)
**Has promoted the portal to a patient, n (%)**			
	Yes	391 (42.5)	
	No	528 (57.5)	

### Factors Associated With Support for the Portal Implementation

The results of the regression analysis (Table 2) revealed associations between the independent variables and the personnel’s support for the patient portal. Thus, Hypotheses 1-3 were supported. The results of the univariate linear regression models (not shown in Table 2) indicated that the personnel’s perceptions of their units’ implementation practices (*β*=.12, *P*<.001), usability of eHealth tools (*β*=.20, *P*<.001), and their eHealth-related competence (*β*=.21, *P*<.001) were positively associated with their support. These variables together explained 17% of the variation in the support for the portal (Model A). The associations persisted after adjustments for age, gender, work experience, organization, and profession (Model B).

**Table 2:** Linear regression results showing association between independent variables and health care personnel’s support for patient portal.

Variables		Model A		Model B	
		*β* (standard error)	*P* value	*β* (standard error)	*P* value
Implementation practices		.07 (0.02)	<.001	.06 (0.02)	.003
Usability		.11 (0.02)	<.001	.10 (0.02)	<.001
eHealth competence		.15 (0.02)	<.001	.15 (0.02)	<.001
Age		—^a^	—	.04 (0.03)	.12
Gender (Category reference: Man)		—	—	–.07 (0.07)	.26
Work experience		—	—	–.04 (0.02)	.13
Organization (Category reference: A)		—	—	–.12 (0.05)	.01
**Profession (Category reference: nurse/midwife/public health nurse)**		—	—		
	Assistant/other nurse			–.01 (0.05)	.79
	Ward/department secretary			–.04 (0.09)	.66
	Social worker			–.03 (0.09)	.77
	Doctor/dentist			.10 (0.10)	.33
	Psychologist/physio and other therapist			.09 (0.09)	.30
	Maintenance and technical support			–.17 (0.11)	.12
	Administrator			.15 (0.08)	.06
	Dental nurse or hygienist			.10 (0.14)	.45
	Other			–.06 (0.13)	.67
R^2^		0.17	—	0.17	—

^a^Not available.

### The Association Between Support and Promotion to the Patients

Table 3 presents the results of the logistic regression regarding the association between personnel’s support for and promotion of the portal to patients. Hypothesis 4 was supported. The personnel’s support was positively associated with their promotion of the portal, and the association persisted after adjusting for age, gender, work experience, organization, and profession. The odds ratio regarding support for the portal was 1.18 after adjustments. This suggests that 1 SD improvement in support was associated with an 18% increase in the likelihood of promoting the portal to patients. Assistant nurses, social workers, doctors, and therapists were less likely to have promoted the portal to patients than were nurses.

**Table 3:** Logistic regression results showing predictors of promotion of the portal to patients, odds ratios, and 95% CIs.^a^

Predictors			Model A					Model B				
			Odds ratio (95% CI)		*P* value		Odds ratio (95% CI)			*P* value		
Support for patient portal			1.22 (1.07-1.40)		.004		1.18 (1.02-1.38)			.03		
Age			—^b^		—		1.13 (0.94-1.36)			.19		
**Gender**			—		—							
	Man					1			—			
	Woman					1.02 (0.63-1.65)			.95			
Work experience			—		—		1.01 (0.84-1.21)			.90		
**Organization**			—		—							
	A					1			—			
	B					0.48 (0.34-0.68)			<.001			
**Profession**			—		—							
	Nurse/midwife/public health nurse					1			—			
	Assistant/other nurse					0.22 (0.15-0.32)			<.001			
	Ward/department secretary					0.80 (0.43-1.48)			.48			
	Social worker					0.41 (0.23-0.75)			.004			
	Doctor/dentist					0.32 (0.16-0.62)			.001			
	Psychologist/physiotherapist and other therapist					0.31 (0.16-0.61)			.001			
	Maintenance and technical support					0.35 (0.17-0.73)			.005			
	Administrator					0.54 (0.27-1.06)			.07			
	Dental nurse or hygienist					1.39 (0.41-4.71)			.60			
	Other					0.60 (0.16-2.94)			.44			

^a^Continuous variables were used as continuous standardized variables.

^b^Not available.

### The Factors Associated With Personnel’s eHealth-Related Stress

The regression results (Table 4) revealed the associations between the independent variables and the personnel’s eHealth-related stress. Hypotheses 5-7 were supported. The results of the univariate models indicated that the personnel’s positive perceptions of their units’ implementation practices (*β*=−.22, *P*<.001), IT usability (*β*=−.35, *P*<.001), and their eHealth-related competence (*β*=.21, *P*<.001) were associated with lower levels of eHealth-related stress. These variables explained 23% of the variance in eHealth-related stress (Model A). The associations persisted after adjustments (Model B). Older age was significantly associated with higher eHealth-related stress (*P*=.01).

**Table 4:** Regression results presenting association between independent variables and personnel’s eHealth-related stress.

			Model A					Model B				
			*β* (standard error)		*P* value		*β* (standard error)			*P* value		
Implementation practices			–.14 (0.03)		<.001		–.14 (0.03)			<.001		
Usability			–.27 (0.03)		<.001		–.28 (0.03)			<.001		
eHealth competence			–.07 (0.03)		.010		–.06 (0.03)			.02		
Age			—^a^		—		.08 (0.03)			.01		
Gender (Category reference: Man)			—		—		–.01 (0.08)			.94		
Work experience			—		—		.03 (0.03)			.34		
Organization (Category reference: A)			—		—		.09 (0.06)			.12		
**Profession (Category reference: nurse/midwife/public health nurse)**			—		—							
	Assistant/other nurse					–.02 (0.06)			.71			
	Ward/department secretary					.08 (0.11)			.48			
	Social worker					–.21 (0.11)			.07			
	Doctor/dentist					–.11 (0.10)			.30			
	Psychologist/physiotherapist and other therapist					–.32 (0.10)			.002			
	Maintenance and technical support					.06 (0.11)			.59			
	Administrator					–.15 (0.14)			.29			
	Dental nurse or hygienist					–.29 (0.17)			.09			
	Other					–.24 (0.22)			.27			
R^2^			.22		—		.27			—		

^a^Not available.

## Discussion

### Principal Findings

Successful implementation of patient portals depends on HCP’s adoption of the portals in their daily work routines. This survey study among primary HCP set out to explore the factors associated with their support for a patient portal and the adverse effect of the portal on HCP in terms of increased eHealth-related stress.

In this survey study with a representative and rather large sample of responses (n=919) from the primary care personnel, we found support for all of our 7 hypotheses. First, in line with a previous study from the preimplementation phase of a patient portal [15], we found that HCP’s perception of the good implementation practices in their unit was associated with their high support for the patient portal. Second, consistent with several previous findings [33-35], an association between good usability and portal support was found.

Third, we found a positive association between HCP’s eHealth competence and their support for the patient portal. We are not aware of previous evidence on this association in the eHealth context. However, in the context of quality improvement in health care, Damush and colleagues [49] found that professionals’ confidence in their ability to perform behaviors required in the new practice was crucial for their acceptance of the improvement.

Fourth, this study shows an association between HCP’s support and their endorsement of the portal to the patients. Previous studies have elaborated on a plethora of factors that inhibit and facilitate professionals’ endorsement [10,50]. However, this study is to our knowledge the first to show quantitative evidence of the association between professionals’ support for the portal and their endorsement of it to patients.

Fifth, in this study, good usability and high self-rated eHealth competence were found to alleviate eHealth-related stress. This is in line with previous studies showing that usability issues increase stress related to information systems, and that these issues may be alleviated by experience in using the technology [24,25].

Finally, we found that, in addition to good usability and high eHealth competence, HCP’s perception of good implementation practices applied in their unit alleviate HCP’s eHealth-related stress. To our knowledge, this association has not been examined before in the eHealth context. Similar associations between implementation practices and employee stress concerning new technology have, however, been found in other organizational contexts [51,52].

### Strengths and Limitations

This study contributes to the research on patient portals by providing quantitative evidence of the roles of (1) managerial practices in the successful implementation, and (2) HCP’s support toward a patient portal. First, studies on good eHealth implementation practices have relied mainly on qualitative evidence [19]. This study aimed to quantify the influence of these practices. While the association between good implementation practices and HCP’s support for eHealth services has been shown before [15], this study suggests that the same practices may also alleviate eHealth-related stress experienced by the personnel. Second, HCP’s positive attitude toward the patient portal was measured with items reflecting respondent’s personal and perceived collective support for new HIT. This concept of user’s positive attitude is not limited to the act of using specific features of the portal but extends to the attitudes toward broader and longer-term changes that the implementation is perceived to entail. We propose that this concept is well-suited to depict HCP’s attitudes in a context where the solution is not expected to remain unchanged but rather to be iteratively developed and molded in a social process to serve the purposes shared by the user, co-workers, and supervisors.

The limitations of this study are related to the cross-sectional, single-informant design, and omission of likely influential contextual factors in the regression models. First, this study relied on self-reported measures. This could lead to problems associated with common method variance and the inflation of the strength of relationships. To minimize the problems with self-reports, measures that exhibited good reliability in previous studies were applied. Second, as we wanted to keep the questionnaire at a suitable length, we were not able to include all relevant contextual factors in our analysis. This is a limitation especially in our model predicting professional support, in which relatively low proportion of variance (17%) could be explained. In particular, the survey did not include questions on the perceived usefulness of the patient portal. Although the functionalities in the studied patient portals were similar to those adopted by other Finnish health care organizations and represented well the functionalities that patients consider useful [48,49], it is possible that personnel’s perceptions of their utility in patient care varies. Studies show that clinicians’ perceptions of HIT utility in terms of improvements in patient care and personnel’s work are among the most important factors in their support [32,50].

The data for this study were collected in 2017. In the field of fast developing eHealth, a delay in reporting the findings on a specific technological application may compromise the timeliness of the observations [53]. However, issues related to users’ adoption of new IT seem to persist over time in the different contexts of technological applications. For example, in Finland, health care professionals’ stress related to information systems has been shown to increase over time regardless of the changes in the applications [44]. We therefore maintain that our findings on the factors that contribute to professionals’ support and stress related to eHealth endure over time and can be generalized to adoption and use of patient portals with different features than what was studied here.

### Practical Implications

The findings of this study have several implications for health care managers and frontline leaders. In order to build HCP support and alleviate employees’ stress related to new HIT, management needs to ensure good usability of eHealth tools, engage personnel in the planning, and provide adequate information on the tools and resources to normalize their use in the daily practices. In the procurement of new HIT, managers are encouraged to acknowledge the proper implementation as a prerequisite for meaningful use. The findings call for careful consideration of the resources needed in the adoption and maintenance phases to balance the investments required and the pace of adoption of new HIT. Experiences beyond the health care industry show that the investments in the implementation and normalization of new HIT are often overlooked in the procurement of new HIT [54].

### Conclusions

This survey study suggests that health care organizations’ implementation practices and good usability have a twofold impact on meaningful and sustainable use of patient portals: first, health care professionals’ stress is relieved, and second, their support for the patient portal increases. Higher support is manifested in professionals’ increased endorsement of the portal to the patients.

## Appendix

Multimedia Appendix 1 Questionnaire.

Multimedia Appendix 2 The means and standard deviations of the study variables.

## Abbreviations

HCP: health care personnel
HIT: health information technology
IT: information technology
UMUX: Usability Metric for User Experience
