# Supplementary material for: Building Primary Health Care Personnel’s Support for a Patient Portal While Alleviating eHealth-Related Stress: Survey Study
Source: J Med Internet Res. 2021 Sep 22;23(9):e28976. doi: 10.2196/28976 (PMC8495577; doi:10.2196/28976)
Supplement: Multimedia Appendix 1 [file jmir_v23i9e28976_app1.docx]

**Multimedia appendix 1. The questionnaire**

The scales for eHealth competences, implementation practices, support and usability ranged from from 1 (fully disagree) to 4 (fully agree) and included a fifth option, 5 (I don’t know).

**eHEALTH COMPETENCES [37]**

**Please evaluate your competence in the following:**

1. I have good basic computer skills.
2. I can work according to the principles of information security and privacy protection.
3. I can use eHealth applications and services.
4. I can utilize the professional’s section of the patient portal.
5. I can utilize the professional’s materials from Kanta.fi*
6. I can fluently communicate with patients via a computer
7. I can advance the implementation of a new eHealth service.
8. I can redesign my own work processes related to eHealth.

*Note: Kanta.fi is a national digital patient data repository that also includes instructions and information for healthcare professionals.

**ORGANIZATION’S IMPLEMENTATION PRACTICES [15, 39]**

**Please evaluate the implementation practices of new eHealth services in your unit:**

1. The personnel receives enough information about new services before implementation.
2. The personnel has a possibility to participate in planning of new services.
3. There is enough training available for using new services.
4. The personnel is allowed to have separate working time for adopting new services.

**SUPPORT [15, 39-41]**

**What is your opinion of the patient portal services?**

1. I support the use of the patient portal.
2. My co-workers support the use of the patient portal.
3. My supervisors support the use of the patient portal.
4. I adapt well to the use of the patient portal.
5. I understand our organization’s decision to implement the patient portal.

**USABILITY [15, 42]**

**Please evaluate the current information technology in use (e.g. information systems and programs, eHealth services, and equipment):**

1. The information technology meets my requirements.
2. The use of information technology is frustrating.
3. The information technology is easy to use.
4. The use of the information technology is enjoyable.

**STRESS RELATED TO eHEALTH AND INFORMATION SYSTEMS [43-45]** (five-point Likert scale ranging from 1 (never) to 5 (very often))

**How often (during the past three months) have you been distracted by, worried about, or stressed about:**

1. constantly changing information systems
2. eHealth services
3. difficult, poorly performing IT equipment/software

**PROMOTION TO PATIENTS** (Scale with options “Never”, “1-4 times”, “5-9 times” and “10 or more times”

**Have you promoted eHealth services to the patients?**

I have promoted the use of the patient portal services to the patients.
