# Supplementary material for: Building Primary Health Care Personnel’s Support for a Patient Portal While Alleviating eHealth-Related Stress: Survey Study
Source: J Med Internet Res. 2021 Sep 22;23(9):e28976. doi: 10.2196/28976 (PMC8495577; doi:10.2196/28976)
Supplement: Multimedia Appendix 2 [file jmir_v23i9e28976_app2.docx]

**Multimedia appendix 2: The means and standard deviations of the study variables**
